# Disparities in Hepatocellular Cancer Screening in Cirrhotic Patients With Psychiatric Disorders

**DOI:** 10.7759/cureus.23516

**Published:** 2022-03-26

**Authors:** Sherif Saleh, Abdul Mohammed, Josue Davila, Neethi Paranji, Bolin Niu

**Affiliations:** 1 Internal Medicine, MetroHealth Medical Center, Cleveland, USA; 2 Hospital Medicine, Cleveland Clinic Foundation, Cleveland, USA; 3 Gastroenterology and Hepatology, MetroHealth Medical Center, Cleveland, USA

**Keywords:** cancer screening, cirrhosis, disparities, psychiatric illness, hepatocellular carcinoma

## Abstract

Background

Patients with psychiatric disorders are at an increased risk of developing liver diseases, including hepatocellular carcinoma (HCC). HCC is a leading cause of cancer-related deaths in the United States. The aim of this study was to re-examine the association of psychiatric illness with HCC and assess its impact on screening practices and the outcomes of HCC.

Materials and methods

We performed a retrospective manual chart review of all patients diagnosed with HCC at a major safety-net hospital in Cleveland, Ohio, from January 2010 to December 2019. Patients were divided into two groups, those with and those without psychiatric illness. The patient characteristics recorded included psychiatric illnesses, etiology of liver disease, radiographic screening intervals, and tumor board recommendations upon initial diagnosis. We analyzed data using Statistical Product and Service Solutions version 26.0 (IBM Corp., Armonk, NY). We analyzed the qualitative and quantitative differences between the groups using the chi-square or Fisher’s exact tests for categorical variables and t-test for continuous variables.

Results

There were a total of 393 patients with a diagnosis of HCC. Among them, 128 (32.5%) were diagnosed with at least one psychiatric illness. Fewer patients with psychiatric illness (33.6%) underwent screening within six months before being diagnosed with HCC compared to those without psychiatric illness (49.8%) (p = 0.002). Patients with psychiatric illness (71.1%) were more likely to have been seen by a gastroenterologist or hepatologist before their diagnosis of HCC compared to those without psychiatric illness (55.1%) (p =0.002). Patients with psychiatric illness were more likely to be offered systemic chemotherapy or hospice (39.1%) compared to those without psychiatric illness (29.1%) (p =0.039).

Discussion

A significant number of HCC patients in our study group have an underlying psychiatric illness. Patients with psychiatric disorders are prone to high-risk behaviors, likely predisposing them to chronic liver disease and HCC. Patients with psychiatric disorders are less compliant with screening practices. Our findings suggest that psychiatric illnesses tend to be diagnosed with more extensive HCC, which is less amenable to curative treatment. Significant efforts need to be made to identify barriers to HCC screening in cirrhotic patients with psychiatric disorders.

## Introduction

In the United States (US), the incidence of hepatocellular carcinoma (HCC) has been exponentially rising over the last two decades while the incidence of other primary cancers of the lung, breast, prostate, and colon are decreasing [1]. HCC patients have a five-year survival of less than 30%, and HCC is the fifth leading cause of cancer deaths in the US [2-3]. While a high prevalence of psychiatric illnesses is reported in cancer patients, very few studies exist that have evaluated the relationship between psychiatric illness and HCC screening [4-7].

There is an association between psychiatric disorders and liver disease. A propensity score-matched analysis of patients derived from the Surveillance, Epidemiology, and End Results (SEER) database and the Medicare Health Outcomes Survey (MHOS) reported poor mental health outcomes in those with HCC diagnosis [8]. Recent reports indicate that concurrent psychosocial factors can influence hepatic physiology and reserve [4-8]. Fuller et al. showed that mental illness is a significant risk factor in the development of liver disease in veterans [9].

Professional society guidelines recommend semi-annual screening for hepatocellular cancer in patients with cirrhosis [10]. Implementation of guideline-driven HCC screening in cirrhosis patients with abdominal ultrasound every six months leads to earlier tumor stage at diagnosis, improved HCC treatment options, and higher survival [10]. We aim to investigate the relationship between the presence of psychiatric illness and adherence to HCC screening. Secondarily, we hope to elucidate any difference in tumor stage at diagnosis and receipt of treatment between those with and without psychiatric illness.

## Materials and methods

Study design

This retrospective study was performed by conducting a manual review of electronic health records of patients diagnosed with HCC at MetroHealth Medical Center, a major safety-net hospital in Cleveland, Ohio, between January 2010 and December 2019. The study protocol was approved by the MetroHealth Medical Center/Case Western Reserve University Institutional Review Board.

Subjects

Patients diagnosed with HCC, as confirmed by imaging or pathologic criteria, between January 2010 and December 2019, were included in the study. Patients younger than 18 in age were excluded from the study. Psychiatric illness or disorders were defined as major depressive disorder (MDD), generalized anxiety disorder (GAD), schizophrenia, bipolar disorder, and other personality disorders. The patients were categorized into two groups: with and without a psychiatric illness. Baseline characteristics were recorded, including age, gender, race, ethnicity, patient insurance, and etiology of HCC. HCC screening intervals were determined as having an ultrasound, computed tomography (CT), or magnetic resonance imaging (MRI) within or more than six months prior to the diagnosis of HCC. Other variables recorded were encounters with gastroenterology or hepatology before HCC diagnosis, alpha-fetoprotein (AFP) levels at diagnosis, model for end-stage liver disease (MELD) score at diagnosis, tumor inside or outside of Milan criteria at the time of diagnosis, metastases at diagnosis, and tumor board recommendations upon diagnosis of HCC. Mortality was defined as death from any cause within 10 years of HCC diagnosis.

Statistical analysis

All statistical analyses were performed using Statistical Product and Service Solutions version 26.0 (IBM Corp., Armonk, NY). Continuous variables were expressed as mean +/- standard deviation or median (interquartile range (IQR)). Categorical variables were presented as numbers (percentage). Qualitative and quantitative differences between groups were analyzed by the chi-square or Fisher’s exact tests for categorical variables and the student’s t-test for continuous variables. A p-value of < 0.05 was recorded as statistically significant.

## Results

Patient characteristics

We identified 393 patients with HCC between January 2010 and December 2019. Among them, 128 (32.5%) patients had at least one psychiatric diagnosis while 265 (67.5%) patients had no psychiatric diagnosis (Table 1). The mean age of HCC patients with psychiatric illness was 60 ± 7.2 as compared to 61.9 ± 9.0 in the non-psychiatric group (p=0.041). There was no significant difference in gender (p=0.127), race (p=0.216) or ethnicity (p=0.473), or type of medical insurance (p=0.174) between the groups.

**Table 1:** Demographics and patient characteristics of patients with HCC and a psychiatric disorder compared to those without a psychiatric disorder HCC: hepatocellular carcinoma; HBV: hepatitis B; HCV: hepatitis C; NAFLD: nonalcoholic fatty liver disease

	HCC total (n=393)	HCC with a psychiatric diagnosis ( n=128)	HCC without a psychiatric diagnosis (n=265)	P-value
Age (mean ± sd)	61.3 ± 8.5	60 ± 7.2	61.9 ± 9.0	0.041
Gender				0.127
Male	318 (80.9%)	98 (76.6%)	220 (83.0%)	
Female	75 (19.1%)	30 (23.4%)	45 (17.0%)	
Race				0.216
African American	163 (41.5%)	49 (38.3%)	114 (43.0%)	
White	182 (46.3%)	64 (50.0%)	118 (44.5%)	
Asian	9 (2.3%)	1 (0.7%)	8 (3.0%)	
Hispanic ethnicity	37 (9.4%)	14 (10.9%)	23 (8.7%)	0.473
Insurance				0.174
Medicaid	186 (47.3%)	63 (49.2%)	123 (46.4%)	
Medicare	138 (35.1%)	40 (31.3%)	99 (37.3%)	
Financial Assistance	22 (5.6%)	11 (8.6%)	10 (3.7%)	
Private	27 (6.9%)	6 (4.7%)	19 (7.2%)	
No insurance	28 (7.1%)	7 (5.5%)	21 (7.9%)	
Liver disease				0.782
HBV	31 (7.9%)	8 (6.2%)	23 (8.7%)	
HCV	311 (79.1%)	103 (80.5%)	208 (78.5%)	
Alcohol	189 (48.1%)	66 (51.6%)	125 (47.2%)	
NAFLD	31 (7.9%)	8 (6.2%)	23 (8.7%)	
HBV status				0.097
HBV on antiviral	12 (38.7%)	5 (62.5%)	7 (30.4%)	
HBV not on antiviral	19 (61.3%)	3 (37.5%)	16 (69.6%)	
HCV status				0.169
HCV treated	111 (28.2%)	42 (32.8%)	68 (33.8%)	
HCV not treated	200 (50.9%)	61 (59.2%)	139 (52.5%)	

Risk factors for HCC

There was no difference in the etiology of HCC between those with or without psychiatric illness (p=0.782). The most common etiology was chronic hepatitis C virus (HCV) infection (80.5% vs. 78.5%), followed by alcohol abuse (51.6% vs. 47.2%), chronic hepatitis B virus (HBV) infection (6.2% vs. 8.7%), and non-alcoholic fatty liver disease (NAFLD) (6.2% vs. 8.7%). There was no significant difference in the status of HBV treatment (p=0.097) or HCV treatment (p=0.169) between both groups (Table 1).

Screening intervals and healthcare encounters before HCC diagnosis

Patients with psychiatric illness were more likely to have been seen by a gastroenterologist or hepatologist before the diagnosis of HCC (71.1%) compared to those without psychiatric illness (55.1%, p=0.002). However, fewer patients with psychiatric illness (33.6%) underwent screening within six months before being diagnosed with HCC compared to those without psychiatric illness (49.8%, p=0.002) (Table 2).

**Table 2:** Screening intervals before HCC diagnosis and healthcare encounters in patients with and without a psychiatric disorder HCC: hepatocellular carcinoma

	HCC total (n=393)	HCC with a psychiatric diagnosis (n=128)	HCC without a psychiatric diagnosis (n=265)	P-value
Screening interval before HCC diagnosis				0.002
Less than 6 months	175 (44.5%)	43 (33.6%)	132 (49.8%)	
More than 6 months	218 (55.5%)	85 (66.4%)	133 (50.2%)	
Seen by GI/hepatology before HCC diagnosis				0.002
Yes	236 (60.0%)	91 (71.1%)	146 (55.1%)	
No	156 (39.7%)	37 (28.9%)	119 (44.9%)	

Severity of liver disease, metastasis, eligibility for liver transplantation, and AFP levels at the time of diagnosis of HCC

There was no significant difference in MELD scores (12.3±5.3 vs. 12.3±5.9, p=0.493) at diagnosis of HCC. In addition, there was no significant difference in the proportion of patients diagnosed within Milan criteria (44.5% vs. 45.7%, p=0.833), the proportion with metastases at diagnosis (34.4% vs. 28.7%, p=0.251), and AFP levels (35.7 vs. 22.0, p=0.309) at the time of diagnosis between the psychiatric and non-psychiatric groups (Table 3).

**Table 3:** Extent of HCC at the time of diagnosis in patients with and without a psychiatric disorder HCC: hepatocellular carcinoma; MELD: model for end-stage liver disease

	HCC Total (n=393)	HCC with a psychiatric diagnosis (n=128)	HCC without a psychiatric diagnosis (n=265)	P-value
MELD at dx (mean, SD)	12.3, 5.7	12.3, 5.3	12.3, 5.9	0.493
Milan criteria				0.833
Inside Milan	178 (45.3%)	57 (44.5%)	121 (45.7%)	
Outside criteria	215 (54.7%)	71 (55.5%)	144 (54.3%)	
Disease metastasis				0.251
Yes	119 (30.3%)	44 (34.4%)	76 (28.7%)	
No	273 (69.5%)	84 (65.6%)	189 (71.3%)	
AFP at dx (median, IQR)	26.6, 406.7	35.7, 366.1	22.0, 400.6	0.309

Clinical outcomes of HCC

Based on the extent of HCC at diagnosis, those with psychiatric illness were more likely to be recommended a palliative approach by the tumor board compared to those without psychiatric illness (39.1% vs. 29.1%) (Table 4). There was no difference in mortality between both groups, with a mortality rate of 59.4% in the HCC with psychiatric diagnosis group compared to 52.5% in the non-psychiatric group (p=0.421).

**Table 4:** Treatment decision at tumor board and mortality rate for patients with HCC and a psychiatric diagnosis compared to those with no psychiatric diagnosis ^1^includes resection, transplant, ablation, and TACE ^2^includes chemotherapy and hospice/palliative HCC: hepatocellular carcinoma; TACE: transarterial chemoembolization

	HCC Total (n=393)		HCC with psychiatric diagnoses (n=128)	HCC without psychiatric diagnoses (n=265)	P-value
Resection	53 (13.5%)		17 (13.3%)	36 (13.6%)	0.157
Transplant	20 (5.1%)		7 (5.4%)	13 (4.9%)	
Ablation	56 (14.2%)		19 (14.8%)	37 (14.0%)	
TACE	115 (29.3%)		27 (21.1%)	87 (32.8%)	
Chemotherapy	32 (8.1%)		15 (11.7%)	17 (6.4%)	
Hospice/Palliative	95 (24.2%)		35 (27.3%)	60 (22.6%)	
Categorized treatment decisions					0.039
Therapeutic^1^		243 (61.8%)	70 (54.7%)	173 (65.3%)	
Palliative^2^		127 (32.3%)	50 (39.1%)	77 (29.1%)	
Death in follow-up					0.421
Yes	216 (55.0%)		76 (59.4%)	139 (52.5%)	
No	107 (27.2%)		33 (25.8%)	74 (27.9%)	

## Discussion

To our knowledge, this is the first retrospective study to compare HCC screening practices in cirrhotic patients with and without psychiatric illness. The results of our study echo the findings of Solmi et al. regarding disparities in cancer screening in patients with psychiatric illness [11]. Patients with psychiatric disorders were less likely to adhere to guideline-based recommendations for screening for HCC compared to patients without a psychiatric disorder. Additionally, patients with psychiatric disorders were diagnosed with HCC at more advanced stages of the disease and consequently were less likely to receive curative treatment.

Our findings suggest that, as previously demonstrated in the literature, psychiatric patients have an increased risk of developing HCC compared to the general population [4-9]. HCV and alcohol use are major contributors to cirrhosis and the HCC burden worldwide [12]. In our study, HCV and alcohol use were among the most common risk factors for the development of HCC. Long-term follow-up of a large group of patients with chronic HCV found a cumulative five-year frequency of HCC of just over 5% [13]. Due to the sizeable overlap between psychiatric illness and substance abuse disorders, patients with psychiatric illness are at high risk of HCV and alcohol-related liver disease [14]. Highly potent, well-tolerated, interferon-free, direct-acting antivirals (DAAs) caused a paradigm shift in the treatment of hepatitis C [15]. Despite the advent of DAAs since 2011, 50% of our study population remained untreated at the time of HCC diagnosis. The number of individuals with psychiatric illness not receiving treatment for HCC was higher. The increased risk of HCC in psychiatric patients is multifactorial and may be the result of lifestyle choices, side effects from psychotropic medications, and concurrent comorbidities, such as obesity, cirrhosis, and NAFLD, which are more prevalent in this population [10,16-17].

A worldwide meta-analysis of cancer screening practices in patients with psychiatric disorders reported less cancer screening than in the general population [11]. Patients with psychiatric disorders have impairment in social functioning, less effective memory, and less medical care-seeking behavior that contributes to less participation in cancer screening [18]. Because advanced HCC is seldom amenable to curative treatments, it is prudent to detect the tumor early when therapeutic intervention is still possible. Patients with cirrhosis are recommended to undergo screening with an abdominal ultrasound every six months [10]. Screening for HCC in patients with cirrhosis improves outcomes by detecting HCC at earlier stages [19]. In our study, psychiatric patients were more likely to be seen by gastroenterologists compared to the general population, and this was likely a result of institutional efforts and outreach programs. Despite being more likely to be established with a gastroenterologist or hepatologist, those with psychiatric illness were less likely to adhere to HCC screening in our study. As a result, a significant number of HCC patients with psychiatric disorders were diagnosed at advanced stages and were not amenable to curative treatments.

Liver transplantation can be curative in select HCC patients, and MELD exception points are awarded to those patients whose tumors are within the Milan criteria [20]. In our study, the proportion of HCC patients with a psychiatric diagnosis that satisfied the Milan criteria for transplantation was not significantly different compared to those without psychiatric illnesses. Consequently, the proportion of patients undergoing liver transplantation for HCC also did not differ substantially between both groups. Psychiatric disorders are potential contraindications for patients seeking liver transplantation because of drug addiction, therapeutic non-compliance, and limited family or social support [21]. Further, psychiatric disorders are associated with increased rates of allograft loss and all-cause mortality in cases with HCC [22]. We did not evaluate post-transplant outcomes in our study.

Our study had a few limitations. First, it is a single-center retrospective study that relied exclusively on electronic medical record data recorded during routine clinical care. Hence, the data likely represent local disease patterns and not the national or global disease burden. Second, we did not analyze the severity of the patient’s psychiatric history and medication compliance, contributing to patient follow-up rates. Third, the impact of psychiatric interventions on the outcomes of HCC was also not evaluated. Finally, our study hypothesizes that psychiatric patients are diagnosed with more advanced stages of HCC due to inadequate screening and thus are more likely to undergo palliative as compared to therapeutic treatment measures. However, a cohort study design would better evaluate this suggested association.

## Conclusions

A significant number of HCC patients in our study had an underlying psychiatric illness. Patients with psychiatric disorders are prone to high-risk behaviors predisposing to chronic liver disease and HCC. Patients with psychiatric disorders were less likely to have adequate screening for HCC and more likely to undergo palliative as compared to therapeutic treatment decisions. Significant efforts need to be made to identify barriers to HCC screening in cirrhotic patients with psychiatric disorders. Multidisciplinary care involving primary care physicians, psychiatrists, gastroenterologists, and hepatologists can help bridge this gap in HCC screening and diagnosis in cirrhotic patients with psychiatric illness.
